# Comparison of Fast-Track Versus Conventional Surgery Protocol for Patients Undergoing Robot-Assisted Laparoscopic Radical Prostatectomy: A Chinese Experience

**DOI:** 10.1038/s41598-018-26372-x

**Published:** 2018-05-22

**Authors:** Zhichao Huang, Lu Yi, Zhaohui Zhong, Liang Zhu, Hongqing Zhao, Yijian Li, Yeqi Nian, Peng Xu, Yinhuai Wang

**Affiliations:** Department of Urology, The Second Xiangya Hospital, Central South University, Changsha, 410011 Hunan China

## Abstract

Fast-track surgery (FTS), which includes a series of evidence-based adjustments, is expected to reduce complications, relieve surgical stress reaction, accelerate recovery, and shorten hospitalization, as well as improve safety. The aim of this study was to critically evaluate the safety and effectiveness of FTS in Chinese prostate cancer (Pca) patients who underwent robot-assisted laparoscopic prostatectomy (RALP). A retrospective analysis was performed on 73 consecutive Chinese Pca patients who underwent RALP and who were divided into two groups: conventional surgery (CS) and FTS. Preoperative clinical data, intraoperative characteristics, postoperative outcomes and incidence of complications were compared between the two groups. No significant differences in preoperative parameters were observed between the two groups. Compared with the CS group, the FTS group showed a significantly shorter time to first flatus, time to regular diet, postoperative hospitalization time, lower incidence of complications, and lower reactions of postoperative stress and pain. Our study demonstrates that FTS is feasible and safe for Chinese Pca patients undergoing RALP and that it accelerates recovery, attenuates surgical stress response, and reduces morbidity compared to CS.

## Introduction

Prostate cancer (Pca) is the most common cancer of the genitourinary tract in men^1^. Radical prostatectomy (RP) is considered an important treatment option for patients with low-risk and intermediate-risk localized Pca and a life expectancy no less than 10 years. With the widespread application and increasing experience of robot-assisted laparoscopic techniques, robot-assisted laparoscopic radical prostatectomy (RALP) has become an increasingly popular minimally invasive choice for managing localized Pca among urologists. Compared with laparoscopic and open RP, a series of randomized trials and comparative studies have provided sufficient evidence about oncologic and functional results to suggest that RALP is a safe and well-tolerated intervention for the treatment of localized Pca^2–4^.

Fast-track surgery (FTS), also defined as enhanced recovery after surgery, was first performed by Kehlet^5^. The FTS protocol includes a variety of techniques with evidence-based interventions, such as surgical, medical, nursing, anaesthetic, preoperative, intraoperative and postoperative managements. The key aims of these protocols are to reduce intraoperative and postoperative complications and relieve surgical stress response, thereby accelerating recovery, shorting hospitalization and reducing medical costs.

The incidental diagnosis of Pca has increased due to the widespread use of modern imaging modalities and PSA screening. Meanwhile, as a result of its inner features, most Pca patients are elderly people, which means that comorbidities are very common. Therefore, it is necessary for urologists to reduce complications and accelerate recovery from medical treatment. Although recent studies have proven the safety and effectiveness of FTS for Western populations in general and urological surgery^6–9^, there is a lack of data in the current literature about whether the FTS protocol is superior to the conventional protocol for Chinese Pca patients who have undergone RALP. To further evaluate the effectiveness and safety of FTS, we compared perioperative parameters and short-term outcomes of Chinese Pca patients who underwent RALP with FTS or conventional surgery (CS) in our department.

## Methods

The records of 73 men who underwent RALP from October 2015 to November 2017 were retrospectively reviewed. All relevant clinical data were collected, and preoperative evaluation of each patient was performed, including age, body mass index (BMI), PSA level, the biopsy Gleason score, clinical stage, medical history, the American Society of Anesthesiologists (ASA) classification score, and laboratory blood tests. All of the patients enrolled were diagnosed with Pca. According to the different perioperative protocols, all the patients were divided into two groups (the CS group and FTS group). The main principles of the FTS and CS protocols are listed in Table 1.

**Table 1:** Protocols of the conventional and FTS groups.

	CS group	FTS group
Preoperative education	Information about RALP surgery	Detailed information on the FTS protocol, Pca, and RALP surgery
Preoperative bowel preparation	Streptomycin (oral 1.0 g tid 2 d) and metronidazole (oral 0.4 g tid 2 d) were given. Semiliquid diet on the second day before surgery Clear liquid diet on the day before surgery 137.15 g polyethylene glycol electrolyte powder mixed with 2 L water the afternoon before surgery A mechanical bowel preparation was performed the evening before surgery.	Clear liquid diet on the day before surgery 137.15 g polyethylene glycol electrolyte powder mixed with 2 L water the afternoon before surgery No mechanical bowel preparation
Preoperative fasting	A mandatory overnight 12 h fast was performed before surgery.	A mandatory fast starting 6 h before surgery.
Intraoperative fluid infusion	Liberal protocol	Restricted protocol (5 ml/kg/h)
Postoperative fluid infusion	3000–5000 ml the day of surgery followed by 2500–3000 ml for the next 3–4 d	1000–2000 ml per day for 2–3 d
Postoperative mobilization	Based on patient’s desire	Encourage mobilization as soon as possible POD 0: sit up; POD 1: sit out of bed; POD 2: walk
Postoperative analgesia	Opium analgesics were used on demand.	A single dose of parecoxib 40 mg I.V. on the day of surgery followed by 20 mg I.V. every 12 h for the next 72 h
Postoperative diet	Allowed normal liquid and diet after first flatus	Chewing gum after surgery; POD 2: clear liquid diet; POD 3: semiliquid diet; POD 4: normal diet

RALP was carried out by a single team of surgeons using the da Vinci Robotic 3-Arm System (Intuitive Surgical, Sunnyvale, CA, USA) via a transperitoneal approach with either standard lymphadenectomy or no lymphadenectomy^10^. The criteria for hospital discharge included tolerance of a regular diet, normal body temperature, mobilization out of bed, and satisfactory pain control. All complications were classified using the Clavien system.

The study was approved by the Ethics Review Board of Second Xiangya Hospital and conformed to the Declaration of Helsinki. Informed consent was obtained from all participants.

### Preoperative education

In the FTS group, patients were given detailed information on the FTS protocol as well as information about the RALP surgery. In the CS group, we only informed patients about the RALP surgery itself.

### Preoperative bowel preparation and fasting

In the FTS group, all the patients were instructed to consume a clear liquid diet one day before the operation and take 137.15 g polyethylene glycol electrolyte powder mixed with 2 L of water in the afternoon prior to the operation. No mechanical bowel preparation was performed. A mandatory fast was started 6 h before operation. In the CS group, patients were given streptomycin (oral 1.0 g tid 2 d) and metronidazole (oral 0.4 g tid 2 d). On the second day before operation, patients were instructed to have a semiliquid diet. On the day before operation, patients were instructed to consume a clear liquid diet and take 137.15 g polyethylene glycol electrolyte powder mixed with 2 L of water in the afternoon. Meanwhile, a mandatory overnight 12 h fast was carried out before operation, and a mechanical bowel preparation was performed in the evening before the operation.

### Intraoperative and postoperative fluid infusion

In the FTS group, all patients received an intraoperative restricted fluid infusion protocol (5 ml/kg/h) and 1000–2000 ml fluid infusion per day for 2–3 days after the operation. In the CS group, all patients received intraoperative standard fluid therapy, which meant that a total of 3000–5000 ml fluid was infused per patient on the day of the operation. Then, all patients were given approximately 2500–3000 ml fluid per day for the next 3–4 days.

### Postoperative mobilization

In the FTS group, patients were encouraged to ambulate postoperatively as soon as possible. If the patients’ conditions allowed, early mobilization was carried out on postoperative day (POD) 0 and the rest of the PODs (sit up on day 0, sit out of bed on day 1, walk on day 2). However, patient ambulation in the CS group was mainly based on the patient’s own desire.

### Postoperative analgesia

In the FTS group, a single dose of parecoxib 40 mg I.V. on the day of surgery followed by 20 mg I.V. every 12 h for the next 72 h was given to patients. In the CS group, opium analgesics were used on demand.

### Postoperative diet

In the FTS group, chewing gum was used on POD 1^11^. Then, patients were allowed to drink clear liquid on POD 2. On POD 3, a semiliquid diet was given. On POD 4, a normal diet was resumed. In the CS group, patients were only instructed to have a liquid and diet after first flatus.

### Statistical analysis

All statistical analyses were performed using SPSS 17.0 for Windows (SPSS Inc., Chicago). All results are presented as the mean ± standard deviation (SD) and frequency (number of cases). Comparisons between two groups were carried out by Student’s t-test for continuous variables and the Mann-Whitney U test for categorical variables. For all tests, *P* values of <0.05 were considered statistically significant.

## Results

A retrospective analysis of 73 consecutive Chinese patients with localized Pca who underwent RALP in our department between October 2015 and November 2017 was carried out. Of the 73 patients, 37 underwent RALP before the introduction of the FTS protocol (CS group), and 36 underwent RALP after the introduction of the FTS protocol (FTS group). The demographic and preoperative clinical data are shown in Table 2. No significant differences in age, BMI, PSA level, ASA, clinical stage, biopsy Gleason score, white blood cell (WBC) count, haemoglobin (Hb) level, or serum C-reactive protein (CRP) level were observed between the two groups (all *P* > 0.05).

**Table 2:** Preoperative characteristics of the patients.

	CS group (n = 37)	FTS group (n = 36)	*P*
Age (years)*	63.5 ± 7.4 (45–75)	62.1 ± 6.9 (46–74)	0.399
BMI (kg/m^2^)^#^	23.5 ± 2.2 (17.9–27.1)	23.1 ± 2.1 (18.1–26.9)	0.440
PSA (ng/ml)	15.40 ± 10.59 (4.10–45.55)	13.44 ± 8.01 (4.22–44.15)	0.377
Gleason score			0.935
<7	16	17	
3 + 4	8	6	
4 + 3	6	7	
>7	7	6	
Clinical stage			0.415
T1	20	16	
T2	17	20	
ASA			0.663
I	16	14	
II	15	15	
III	6	7	

*Mean ± standard deviation (range); ^#^BMI: body mass index.

Table 3 shows details about the operative outcomes. There were no significant differences in operative time or estimated blood loss between the two groups (both *P* > 0.05). Compared with the CS group, the patients in the FTS group had a significantly shorter postoperative hospital stay, time to first flatus and time to regular diet (all *P* < 0.05). With the purpose of assessing the surgical stress response, the WBC counts and serum CRP levels were measured on PODs 1, 3, 5 and 7 (Table 4). No significant differences in WBC count or CRP level were observed between the two groups at POD 1 or 3 (all *P* > 0.05). However, at PODs 5 and 7, WBC count and CRP levels in the CS group were significantly higher than in the FTS group (all *P* < 0.05). Meanwhile, the postoperative pain control in the FTS group was better than that in the CS group, which meant less additional opium analgesic consumption (*P* = 0.004).

**Table 3:** Intraoperative and postoperative outcomes.

	CS group (n = 37)	FTS group (n = 36)	*P*
Operative time (min)	147 ± 29 (98–251)	135 ± 32 (91–268)	0.801
Estimated blood loss (ml)	179 ± 65 (50–310)	191 ± 64 (55–330)	0.456
Time to first flatus (d)	3.6 ± 1.5 (1.0–6.0)	2.6 ± 1.1 (1.0–5.0)	0.001
Time to regular diet (d)	5.9 ± 2.1 (2.0–10.0)	4.2 ± 1.7 (2.0–10.0)	<0.001
Postoperative hospital stay (d)	9.8 ± 2.0 (6.0–15.0)	7.3 ± 1.6 (5.0–14.0)	<0.001
pain (n)*	15	4	0.004
Complications (Clavien grade I-II, n)	15	6	0.025
Nausea	4	2	
Lymphocele	1	1	
Urinary leakage	2	1	
Ileus	3	1	
Wound infection	1	1	
Urinary tract infection	1	0	
Pneumonia	2	0	
Deep venous thrombosis	1	0	

*Patients who needed opium analgesics.

**Table 4:** Preoperative and postoperative values of WBC and serum CRP.

	WBC (×10^9^/L)		*P*	Serum CRP (mg/L)		*P*
	CS group	FTS group		CS group	FTS group	
Preoperative	6.86 ± 1.38 (4.30–9.12)	6.67 ± 1.52 (4.22–9.72)	0.585	3.52 ± 1.17 (1.09–5.79)	3.33 ± 1.07 (1.22–5.68)	0.482
POD 1	15.16 ± 3.28 (8.48–19.89)	14.82 ± 2.83 (9.47–19.21)	0.638	81.11 ± 12.02 (44.79–98.76)	79.37 ± 11.94 (49.71–101.36)	0.536
POD 3	12.13 ± 2.26 (7.21–15.67)	11.80 ± 2.14 (7.70–16.54)	0.531	57.06 ± 12.20 (27.81–71.33)	55.60 ± 12.61 (25.91–76.93)	0.618
POD 5	9.29 ± 1.51 (6.77–12.56)	7.97 ± 1.23 (5.12–11.31)	<0.001	24.21 ± 5.73 (15.81–43.17)	15.76 ± 4.47 (6.98–30.87)	<0.001
POD 7	8.03 ± 0.93 (5.47–9.61)	6.51 ± 1.02 (4.81–8.47)	<0.001	12.18 ± 2.51 (7.77–16.92)	8.40 ± 2.11 (4.67–12.13)	<0.001

The details of complications in both groups are listed in Table 3. All the complications in both groups were minor complications that were classified as Clavien grade I or II. The complication rates in the CS group were significantly higher than those in the FTS group (*P* = 0.025). There were no cases of conversion to open surgery.

## Discussion

In recent years, there has been a rapid adoption of RALP among urologists for the management of Pca patients. Furthermore, with evidence of improved convalescence, RALP has advantages in minimizing operative injury and reducing complications in comparison to open surgery and laparoscopic RP^3,12^. However, with increasing experience in RALP, a trend has emerged in experienced medical centres to implement the FTS into RALP programmes. The FTS or EARS protocol is a standardized multimodal perioperative care pathway that aims to reduce postoperative morbidity, minimize the negative stress effects of surgery, accelerate recovery and shorten hospital length of stay (LOS). After RALP was first introduced in the 1990s in colorectal surgery, it has been widely implemented in abdominal and gynaecologic surgery^13–15^. However, there is a lack of studies on fast-track programmes in RALP. In the current study, we describe the short-term outcomes of RALP for Chinese Pca patients with FTS or CS in our department. Our results demonstrate that the implementation of FTS obviously accelerated postoperative recovery and was safe and effective.

In the traditional programme, the operation and medication are the keys to the whole treatment. However, in our study, preoperative education played an important role in FTS patients’ learning about Pca, about RALP, about each detailed step of the FTS protocol and about the potential morbidities. The key aims of preoperative education are to help patients relieve their psychological and physiological stress and proactively cooperate with urologists and nurses. A previous study has demonstrated that patient education about FTS, preoperative investigations and relevant information offered is essential for patients to have a better physiological state, a more rapid return to normal quality of life, a shorter stay in intermediate care, and a faster recovery from surgery^16^.

Mechanical bowel preparation accompanied by medication and long-term fasting has been routinely performed in Pca patients undergoing RP to reduce the risk of postoperative infections and morbidities resulting from potential rectal injury during the procedure. However, previous studies have questioned the benefit of bowel preparation^17,18^. Indeed, traditional bowel preparation can lead to some adverse side effects, such as dehydration, electrolyte or acid-base disturbance, and intestinal mucosal architectural change^19,20^. Therefore, we performed an improved one-day bowel preparation in the FTS group. Our results demonstrate that the FTS protocol was associated with significantly more rapid intestinal motility and a shorter period to bowel function recovery without obviously increasing the risk of postoperative complications than with the CS protocol.

Traditionally, the main propose of intraoperative and postoperative fluid infusion is to maintain hydro-electrolyte balance and supply the basic energy needs for metabolism. However, administering excessive fluid causes intestinal oedema, coagulation dysfunction, impaired wound healing, increased interstitial lung water, hypervolemia and cardiopulmonary complications, which can all negatively influence recovery^21^. Therefore, if the patient is normovolaemic and under the careful monitoring of vital signs, the intraoperative and postoperative restricted fluid infusion will not only ease the patient’s pain but also accelerate recovery^22^. Our results indicate that the restricted fluid therapy was safe and feasible.

Early mobilization is beneficial, as it is associated with supportive effects on muscle strength, bowel function, cardiovascular and respiratory function, and psychological well-being and diminishes the cardiopulmonary complications and thromboembolic effects^23,24^. A previous study analysing the impact of preoperative and postoperative exercise compared with FTS mobilization did not show any significant differences in LOS, readmission, complications or mortality in patients undergoing radical cystectomy. However, there were significant differences in the ability to perform personal activities and mobilize between the FTS and conventional groups in the first 7 d^25^. Unfortunately, poorly controlled postoperative pain not only is an adverse side effect of surgery that limits early mobilization but also leads to the stress response accompanying surgical injury itself, with the potential to cause a series of complications. Adequate and appropriate analgesia is essential to early mobilization and reduction of the postoperative stress response^26^. Because of its intrinsic side effects, opioids have the potential to cause nausea, vomiting and intestinal ileus^26^. Therefore, polypharmacological opioid-sparing analgesia is recommended, and the basic drug should include an NSAID^27^. Parecoxib, an injectable selective cyclooxygenase enzyme-2 (COX-2) inhibitor, can not only relieve postoperative pain but also reduce the postoperative stress response^28^. In the current study, the degree of postoperative stress response was evaluated by measuring the WBC count and serum CRP level. Patients in the FTS group were encouraged to mobilize early, and parecoxib was used in the FTS group instead of the opioid given to the CS group. Our results reveal that the FTS protocol facilitated opiate minimization, reduction of the stress response, and lowered the risk of cardiopulmonary and coagulation complications.

Traditionally, patients undergoing surgery were only allowed to intake liquid after the first flatus passed because the early resumption of oral nutrition was considered to increase the incidence of intestinal complications. Previous studies have demonstrated that early oral intake can not only stimulate gastrointestinal pathways to promote bowel movement but also positively affect the brain-gut axis to facilitate higher cognitive function, such as emotion and decision making, which are associated with a decreased paralytic ileus, fewer infectious complications, increased patient satisfaction, shorter LOS, and a faster recovery^29–32^. Thus, early oral intake is recommended as a key component of the FTS protocol. However, postoperative nausea, vomiting and ileus are the risks of early oral intake. Cephalic-vagal stimulation from gum chewing promotes propulsive and hormonal gastrointestinal activity similar to what appears with normal eating. Gum chewing is a form of sham feeding that can stimulate bowel motility through neurohumoural reflexes^11^. In this study, early oral intake and chewing gum were applied in the FTS group. In concordance with previous studies, all early oral intake and gum chewing were safe and were associated with a lower incidence of complications, minimal problems of tolerance, decreased fluid infusion, and a faster recovery.

There are several limitations to the analysis reported in this study. First, it was a retrospective study with a small sample size in a single clinical centre and not a randomized controlled trial. Thus, the small sample size and the chosen P value may be associated with inflation of the type I error. Due to the statistical nature of a hypothesis test, all statistical results have a probability of inducing type I and type II errors. The only way to reduce both error rates is to increase the sample size. Additionally, the duration of follow-up was not enough to evaluate the long-term outcomes of the FTS protocol. Finally, because the FTS protocol aimed to standardize every phase of preoperative, intraoperative, and postoperative management to enable patients to recover faster, the protocol should be applied by a standardized team consisting of urologists, anaesthetists, nurses, and physiotherapy staff. Further studies with a randomized controlled design, adequate sample size, long-term follow-up, and more than one centre are required to illustrate the value of FTS protocols in RALP.

In conclusion, the results of this study indicate that the introduction of this FTS protocol for Chinese Pca patients undergoing RALP was safe and was associated with reduced stress response, accelerated recovery and shorter LOS.
